# Molecular and Cellular Alterations in Down Syndrome: Toward the Identification of Targets for Therapeutics

**DOI:** 10.1155/2012/171639

**Published:** 2012-07-12

**Authors:** Nicole Créau

**Affiliations:** Unité de Biologie Fonctionnelle et Adaptative (BFA), Sorbonne Paris Cité, Universite Paris Diderot, EAC4413 CNRS, 75205 Paris Cedex 13, France

## Abstract

Down syndrome is a complex disease that has challenged molecular and cellular research for more than 50 years. Understanding the molecular bases of morphological, cellular, and functional alterations resulting from the presence of an additional complete chromosome 21 would aid in targeting specific genes and pathways for rescuing some phenotypes. Recently, progress has been made by characterization of brain alterations in mouse models of Down syndrome. This review will highlight the main molecular and cellular findings recently described for these models, particularly with respect to their relationship to Down syndrome phenotypes.

## 1. Introduction

Down syndrome (DS) is the most frequent human aneuploidy (1/800 births). DS is characterized, in part, by cognitive impairment, which is present to some degree of severity in all affected individuals [1], and by neuropathological alterations similar to those observed in the brains of Alzheimer's disease patients (over 40 years in DS) [2–4]. Specific deficits of the nervous system in DS individuals affect learning, memory, language, and movement [5–8]. These deficits are associated to alterations in volume, in grey matter density and altered neuronal circuits of different regions of the brain [9–13]. DS typically results from the presence of three complete copies of human chromosome 21 (trisomy 21, T21) [14]. Due to the presence of this extra copy of chromosome 21 (HSA21), DS phenotypes are expected to be associated with a gene dosage effect: genes on HSA21 are present in three copies rather than two, leading to 50% overexpression (or 1.5-fold expression levels). Transcriptome and proteome studies have shown that, indeed, a global gene dosage effect is present; however, interestingly, expression of a number of trisomic genes varies: some are compensated (near 1), while others are underexpressed (less than 1) or highly overexpressed (more than 1.5). These changes may vary depending on the cellular component and likely result from gene or protein interactions in pathways or in protein complexes (e.g., complex subunits). These variations have been observed in T21 as well as in different organs of mouse models of DS and as a result of aging [15–21]. Thus, defining which HSA21 genes (or murine orthologs) are particularly responsible for disease phenotypes is crucial: identifying the molecular and cellular variations in conjunction with overexpression will help determine their associations with the phenotype and aid in testing potential molecules for phenotypic rescue.

## 2. Mouse Models of DS

**Figure 1:**
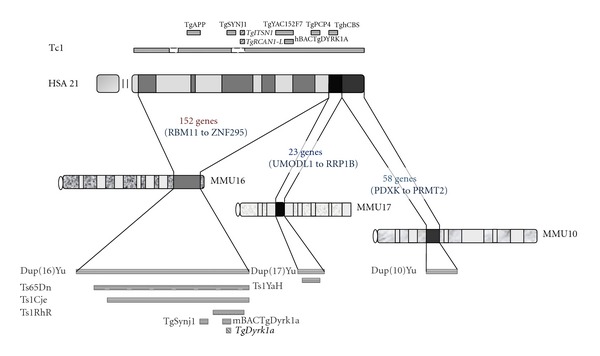
HSA21 (with main cytogenetic bands) and its ortholog segments in the mouse genome (MMU16, MMU17 and MMU10) are indicated. Main mouse models and those reported in this review are indicated in black for human genes, and in gray for mouse genes. Models with cDNA constructs are indicated in italics. Representation of their localisation is not to scale. Tc1 [32]; TghAPP [33]; TgSYNJ1 [54]; *TgITSN1* [55]; *TgRCAN1*-*L* [56]; TgYAC152F7 [34]; hBACTgDYRK1A [24]; TgPCP4 [38]; TghCBS60.4 [57]; Ts65Dn [22]; Ts1Cje [23]; Ts1RhR [58]; TgSynj1 [54]; *TgDyrk1a *[59]; mBACTgDyrk1a [25]; Dup(16)Yu, Dup(17)Yu and Dup(10)Yu [29, 31]; Ts1Yah [30].

Mouse models have been critical to our understanding of the molecular genetics of DS. Several models have been constructed: some have an additional copy of a chromosome segment orthologous to HSA21 [22, 23], others have additional copies of individual genes from HSA21 or their mouse orthologs [24, 25]. Though more recent models have increased our understanding of the consequences of adding one copy of a specific gene or a segment containing multiple genes, the most extensively studied models are the Ts65Dn [22] and Ts1Cje [23] mice, which carry large segmental trisomies for mouse chromosome 16 (MMU16) (Figure 1). These models recapitulate several phenotypes of DS, including reduced brain volume, significant learning and memory impairment, and altered synaptic plasticity measured in hippocampal long-term potentiation (LTP). However, recent investigations into the exact gene composition of these models have shown that, in addition to the duplicated segment of MMU16, Ts65Dn, which results from a translocation onto MMU17, contains a duplication for proximal genes of MMU17 and Ts1Cje contains a deletion of a 7-gene span of MMU12 [26–28]. More recent trisomic models [29–31], constructed using the Cre/loxP-mediated chromosome engineering strategy, have integrated only segments of the mouse chromosomes orthologous to HSA21—MMU16, MMU17, and MMU10 (Figure 1)—eliminating any potential confounding effects from additional genetic aberrations. Another model, Tc1, is a transchromosomic model transmitting a copy of a portion of HSA21 spanning over 75% of the original chromosome [32]. The human genes present on this chromosome are, indeed, expressed in the mouse, confirming that specific models may bear either a human or mouse gene, as previously demonstrated with YACs containing human genes [33, 34]. Interestingly, the Tc1 model may also be useful for evaluation of effects of T21 mosaicism because the transchromosome appears to be retained in only 50–60% of Tc1 adult brain cells [35]. In fact, Papavassiliou et al. [36], in studying the rate of T21+ cells in the buccal mucosa and lymphocytes of individuals with T21 mosaicism, found a positive correlation between patient IQ range and percentage of T21+ cells in their tissues. Thus, the presence of trisomy in at least 50% of brain cells may have strong implications for cognitive development.

Transgenic models introducing a specific HSA21 gene or murine ortholog, and for which molecular and cellular studies have been performed, are presented along with trisomic models in Figure 1 and Tables 1 and 2. Tables 1 and 2 summarize the main studies identifying molecular and cellular changes in these models.

## 3. DS Transcriptome

Gene expression studies have provided much-needed insight into global expression changes occurring in DS. In particular, microarrays have been employed to determine the transcriptome of cells and even brain structures. Ts1Cje and Ts65Dn transcriptomes were analyzed at various developmental timepoints (see Table 1). Changes in transcript levels were observed for genes in three copies, mirroring copy number (i.e., near 1.5-fold). However, more specific analyses of expression changes, as in the cerebellum of Ts65Dn [44], suggest that the genetic backgrounds of trisomic mice may impart individual variations onto expression changes. Further, such inter-individual variations are observed at the protein level [52]. Interestingly, in Tc1 mice genes from HSA21 are expressed at embryonic day (E) 14.5, indicating that these genes are transcribed during mouse embryonic development [32]. Recall that this model leads to a mosaic composition of adult organs with cells containing or not HSA21, detecting the molecular consequences in adults at the transcriptional level may be more difficult. Interestingly, Tc1 mice have impaired short-term memory but normal long-term memory [35]; both features are affected in Ts65Dn mice [53]. These contrasted phenotypes in adult mice suggest that part of the functional alterations in DS results from strong modifications in proliferation and/or differentiation steps of neural components of various brain structures—processes that are established during embryogenesis. Notably, however, the absence of several genes on the human chromosome transmitted in Tc1 may also influence the functionality of the adult brain (Figure 1).

More recent evidence indicates that alternative splicing may play a role in differentiating the brain transcriptome in DS mouse models as well as in DS. Proteins involved in the splicing machinery are modulated and alternative exons of key synaptic transcripts (neuroligins, TrkB, AChE, Mapt) can be expressed, suggesting a different control of the transcriptome in the disease state. Modulated splicing factors (ASF, Srp55, Srp75, Srp30, SC35) were identified at the global protein level or at the phosphorylation level depending on the brain regions explored as well as a result of aging. Notably, at least one HSA21 gene appears to be responsible for dysregulation via splicing factor phosphorylation: *Dyrk1A*. This *proline-directed serine*/*threoninekinase* colocalizes with some of these splicing factors and, further, regulates biogenesis of the splicing speckle compartment [60–62]. In adults with DS, *Dyrk1a* overexpression appears related to overexpression of the 3R isoform transcript of *microtubule-associated protein tau* (*Mapt*), which is predominant in neurofibrillary tangles, suggesting a new role for *Dyrk1a* in neuronal degeneration [61, 63–65].

In addition to protein-coding RNAs, several functional RNAs do not lead to the translation into a protein (noncoding RNA). MicroRNAs (miRNA) belong to the small noncoding RNAs class and have been shown acting on the regulation of translation of gene transcripts either by degradation or repression, thus influencing the content of the proteome. Mounting evidence suggests that miRNAs affect brain development and function [66]. Five miRNAs are transcribed from HSA21, three of which are clustered [46]. HSA21 miRNAs (miR-99a, let-7c, miR-125b-2, miR-155, and miR-802) are overexpressed in the DS brain from fetal to adult stages [46, 67, 68]. In the Ts65Dn mouse model, only miR-155 and miR-802 (both in 3 copies) have been found to be overexpressed in brain [69]. The authors found also that the transcription of the *methyl-CpG-binding-protein* (*Mecp2*) gene, which is mutated in Rett syndrome, is decreased. Intracerebroventricular injection of Ts65Dn with antisense RNA for these two miRNAs (antagomirs) normalizes the expression of* Mecp2* and *Creb* (*cyclic AMP responsive element binding protein*)as well as the Mecp2-regulated gene *Mef2c* (increased in Ts65Dn) [69]. Other possible involvement of miRNAs in brain alterations of DS and mouse models require further investigation [70].

## 4. DS Proteome

Alterations in the transcriptome in DS is expected to have direct implications on the proteome. The brain proteome has been studied using different quantification methods, but its modulations are more difficult to approach on a large scale. Quantitative immunohistochemistry is complementary to these approaches, since it can reveal which cells may be more affected by protein expression changes. Indeed, it is necessary to determine whether any fluctuations in protein expression result from changes at the cellular level or changes in the proportion of cells expressing the protein(s). Current research targeting potential pathways have led to an increase in studies identifying the proteome changes within specific brain structures in DS models.

Table 2 recapitulates significant protein changes (up or down) observed in the trisomic and transgenic mice in function of age and brain structures. Interestingly, these data show that the proteins level even in the same mouse model may increase with age (App and Sod1—which are in 3 copies), may depend on the brain structures (Synaptophysin (Syp) up in cortex versus down in hippocampus; Gaba-b receptor 2 (Gabbr2) up in hippocampus versus down in thalamus) or may be increased from early stages to adult (Map2, Ntf3), though all developmental stages are not yet studied.

## 5. Morphological and Cellular Changes in Brain Structures

The universal presence of cognitive impairment in DS has made understanding the structural and cellular changes in the DS brain the focus of much research effort. Reduction in cerebellum volume is a feature of Down syndrome and is recapitulated in Ts65Dn and to a lesser extent in Ts1Cje models. Interestingly, changes in volume or cellular density appear to differ between regions of the brain, suggesting that gene dosage differentially affects brain structure development [9, 10, 89, 90]. Similarly, enlargement of the lateral ventricle, another alteration in brain morphology, has been observed in both DS and mouse models of DS, specifically Ts1Cje, Ts2Cje [91], Ts65Dn, and mBACTg*Dyrk1a* [25].

Cell proliferation is also altered in DS and in mouse models, suggesting a relationship between alterations in volume and altered cell numbers in brain structures. In cortex, hippocampus, and cerebellum, region volume and neuronal populations are affected [58, 92–96]. These defects in proliferation alter the neuron as well as the astrocyte number and percentage. Recently, proliferation impairment in neural cell precursors of Ts65Dn was shown to involve inhibition of the hedgehog pathway [72]. This finding extends those of Roper et al. [97], who linked hedgehog to decreased granular cell progenitor (GCP) production in the cerebellum of Ts65Dn. Sonic hedgehog (*Shh*), produced by the cerebellar Purkinje cell, typically activates GCP proliferation during cerebellar development, but this pathway is defective in DS models. Similarly, a defect in *Shh* mitotic response is present in neural crest progenitors of these mice [98]. Inhibition of the hedgehog pathway can occur through overexpression of a fragment of amyloid precursor protein (*App*, in 3 copies in the Ts65Dn), AICD (App intracellular domain). Through increased binding of AICD to the *Ptch1* (*Patched*, SHH *receptor*) promoter and histone hyperacetylation, *Ptch1 *is overexpressed [72]. However, silencing of *Ptch1* restores proliferation of neural cell precursors. Indeed, AICD has been shown to act as a transcriptional regulator for its own gene (*App*) as well as other genes [99]. Reduced cerebellar volume also occurs in Ts1Cje mice (2 copies of *App*), but to a lesser extent than in Ts65Dn (3 copies of *App*), suggesting that other 3-copy genes contribute to the proliferation defect through the Shh receptor *Ptch1* [26] or other molecules.

Notably, these proliferation defects may be associated with the surprising lack of medulloblastoma and neuroblastoma tumors observed in Down syndrome [100–102]. In DS models, several genes involved in the regulation of the cell cycle, namely, cell-cycle-dependent kinases *p21Cip1* [103]  and *p27Kip1* [104, 105], are differently affected and induce a dysregulation of the cell cycle. These proteins as well as *Ptch1*, the receptor for Shh [72], have been shown to be important players in medulloblastoma induction [106]. Thus, the alteration in neural proliferation, while likely contributing to cognitive impairment in DS, may protect against these type of tumors. Additionally, increased *Dyrk1a* [107] and *Pcp4* [38, 108, 109] expression are associated with premature neuronal differentiation at early embryonic stages, which may also guard against these tumors by driving neurons to a more mature state.

Interestingly, increased dosage of murine *Dyrk1a* leads to an increase in neurons and glial cells in the thalamus VPL-VPN while other structures, like the somatosensory cortex, though increased in volume, do not show any change in the numbers of these cellular components [25]. Thus, proliferation may be differentially affected in particular regions and cell types during development, as has been visualized in the DS brain [10, 110].

Adult neurogenesis occurs at two major sites in the brain: the subventricular zone of the lateral ventricule and the subgranular zone of the dentate gyrus of the hippocampus (human and mouse). Though the physiological relevance of adult neurogenesis is still under debate, it may have strong implication in new acquisition of memory. Adult neurogenesis is impaired in Ts65Dn hippocampus [111] and can be reversed by treatment with fluoxetine, an inhibitor of serotonin (5-HT) reuptake [112]. Recent experiments using the same molecule rescued neurogenesis in Ts65Dn not only in hippocampus but also other structures (striatum, neocortex) and involved the rescue of expression of the neurotrophic factor BDNF [75], which is crucial for neuron survival. Indeed, BDNF levels (RNA and protein) depict a complex situation in DS that may result partly from a newly identified mechanism acting in brains of DS models: regulation of local translation [113]. BDNF RNA levels are decreased in DS and mouse models, but circulating levels of BDNF are higher in DS [42, 114, 115]. In Ts1Cje, increased BDNF release in the hippocampus occurs through different regulators of synaptic local translation, suggesting a more fine-tuned regulation of this neurotrophic factor. Further, the new hypothesis proposed by Troca-Marín et al. [113] of a positive-feedback loop involving BDNF and the Akt-mTOR pathway suggests new avenues for treatment. This type of regulation may involve other molecules important for brain function, as has already been shown for *Dscam* [116]—which occurs in 3 copies in the mouse models—and still needs to be explored.

Other molecules and pathways contributing to DS neuropathology have been extensively studied. For example, Map2, a microtubule-associated protein present in the soma and dendrites of mature neurons, is increased in hippocampus and cingulate cortex of Ts65Dn, independent of age [71, 87]. Map2 immunolabeling reveals thicker, shorter, and less-tapered dendrites in aged Ts65Dn adult neurons. Further, during embryonic cell differentiation in culture, abnormal neurite branching was observed in neurons of fetal T21 [117] and Tc1 [118], combined with an increase in secondary to primary dendrites. Abnormal dendrites have been previously observed during early development in DS cortex; the overdevelopment of dendritic trees in the visual cortex of DS patients at birth, despite dendritic atrophy later during infancy [119, 120], suggests that temporally different mechanisms may contribute to abnormal maturation of neurons in DS. Though different 3-copy genes might contribute to these changing phenotypes [38, 59, 121], the mechanisms of altered cytoskeletal dynamics remain unexplained.

Another neuronal phenotype in DS is the excitation-inhibition imbalance shown to play a central role in brain malfunction; reducing overinhibition represents a current goal for ameliorating cognitive dysfunction [122, 123]. Over-inhibition may result from an increase in inhibitory neurons [80, 95], an increase in inhibitory synapses [124, 125], an increase in efficiency of inhibitory synapses [126], an increase in stimulation of GABAergic ouput neurons [127], or a decrease in these excitatory components [128]. Moreover, in relation to Girk2 overexpression (*Kcnj6 *in 3 copies) which regulates the GABA-B receptor at dendrites, the balance between GABA-B and GABA-A inhibition is altered in Ts65Dn hippocampus [41, 73, 129]. In Ts65Dn cortex, excitatory neurons exist in the same proportions in control and Ts65Dn brains throughout development; interneurons, however, are increased in Ts65Dn brains. Further, these interneurons show an increased excitability in basal conditions [95]. Reducing copy numbers of *Olig1* and *Olig2 *transcription factors required for oligodendrocyte specification and differentiation [130], rescues the number of cortical interneurons of Ts65Dn [95]. Finally, additional circuitries of neurotransmitter release as well as neuropeptide signaling are impaired ([54, 79, 131, 132]; Table 2).

Though the global composition of Ts65Dn synapses does not differ from controls reduced CaMKIIalpha and increased peptide phosphorylation, potentially important for synaptic function, have been found; synaptojanin 1 (Synj1), which is important for synaptic vesicle recovery and is triplicated in Ts65Dn, is also increased [133]. Additionally, spine morphology and spine density differ [134, 135], but the global level of synaptophysin, a marker of presynaptic vesicles, appears reduced [71]. Decreased spine density has been observed in Ts65Dn hippocampus and temporal cortex [135–137]. Further, synapse enlargement is present in hippocampus, with an associated decreased length of spine neck [135]. Similarities are evident in spine morphology between Ts1RhR [58], Ts1Cje, and Ts65Dn, but with increased severity of phenotype with increased number of genes in 3 copies [134, 138]. Moreover, the trisomy in Ts1RhR is sufficient to induce a decreased average in spine density in the fascia dentata [138]. Finally, endocytosis may be altered by increased levels of Itsn1 [55, 139], *Dyrk1A* [140], Synj1 [141], and interaction with other genes in 3 copies [142]. Together, these anomalies may lead to altered synaptic plasticity, as visualized at the level of hippocampal LTP, and likely regulate learning processes.

Glial cells are another structurally and functionally important component of the brain, serving as support and as regulators of synapse connectivity; they are also present at the blood-brain barrier. Glial fibrillar acidic protein (GFAP) is commonly used to identify these cells. During early development in DS hippocampus and frontal lobe, an increase in GFAP-positive cells is observed [143, 144], together with a more mature morphology [144]. This may result from a preference for glial cell production over neuron production, as seen during the differentiation of neural precursor cells [117, 145–148]. An increase in glial cells has been identified in the Ts65Dn hippocampus during early postnatal development [149]. However, in adult Ts65Dn brain, a decrease in GFAP transcript was observed [47]. Moreover, dysfunction of Ts65Dn astrocytes [40] coupled with an increase in beta-catenin in the microvessels of Ts65Dn brain [150, 151], two important components of the brain-blood-barrier, suggest that its function might be altered.

Interestingly, in aged DS brains, a reduced glial cell number has been observed in the cortex [152], and alterations in the morphology of astroglial cells develops with age [153]. Further, increased GFAP in the frontoparietal cortex and hippocampus of aged Ts65Dn mice revealed gliosis [83]. Thus, altered glia may play a role in the modified functionality of brains of DS mouse models. Notably, alterations in Purkinje axons in the cerebella of Ts65Dn have been observed from 10 months of age, while astrogliosis appears later [85, 86]. These results suggest that the Ts65Dn cerebellum is not protected against neuronal degeneration, which may be detected earlier by specific modifications of neuronal properties.

Finally, identification of aging processes related to Alzheimer's disease pathology are under investigation in DS models. APP has been suspected as a major player in this pathology and increased copy number of *APP* in human is associated with Alzheimer's disease [154]. Other genes on HSA21 may either protect against or enhance the effects of the increase in APP [21, 64, 65]. Aged Tc1 mice (18 months) have an increase in tau phosphorylation and neurofibrillary tangles, features not present in young animals. Further, a correlation with the level of Dyrk1A was found, but only in aged mice [65]. In this model, human proteins like APP, SYN1, ITSN1, and RCAN1 may be absent, suggesting they do not play a role in that process [35]. Transgenic mice with a copy of the entire *APP *[33] or *SYNJ1* [54] gene have been already constructed, but mice transgenic for *ITSN1* and *RCAN1* were constructed with heterologous promoters. Thus, although elevated phospho-tau was observed in transgenic *TgRCAN1-L* [56] mice, confirmation in a model with the entire gene is needed to further understand the role of these genes in Alzheimer's disease pathology.

## 6. Genes and Pathways Targeting

Thanks to these rapid advances in understanding the specific brain alterations in DS, therapeutic approaches are being developed. The first therapeutic assay targeted the specific loss of basal brain cholinergic neurons (BFCN) observed after 6 months in Ts65Dn. This specific loss, due to altered transport of nerve growth factor (NGF), was rescued by infusion of NGF [84], demonstrating the potential for phenotype reversal. As excitation-inhibition imbalance has emerged as a strong target, recent approaches have targeted the potential pathways at the roots of the observed over-inhibition. Fernandez et al. [123], by using an inhibitor of the GABA-A receptor (pentylenetetrazole, PTZ), reversed the phenotype of Ts65Dn, confirming that GABA, the major inhibitory neurotransmitter of the central nervous system, is involved. Though multiple approaches are currently being tested (see Table 1), only two recent approaches have tried to identify—on a large scale—correlations between molecular changes and behavioral changes induced by a therapeutic molecule, in adults of DS models.

Braudeau et al. [43, 155] analyzed the transcriptome of mice submitted to memory processing using the Morris water maze paradigm following treatment with an inhibitor of the GABA-alpha5 receptor, the GABA-alpha5 promnesiant inverse agonist (alpha5IA). The GABA-alpha5 receptor (*Gabra5*) is specifically expressed in the hippocampus and, thus, its modulation directly involves hippocampal function. In combination with the expression of early genes, specific 3-copy genes were modulated significantly: 6 transcripts were upregulated (*Kcnj6*, *Sod1*, *Itsn1*, *Hcls*, *Gart*, *Ifnar2*) and 3 were downregulated (*App*, *Kcnj6*,* Sod1*) in Ts65Dn following treatment. Moreover, a set of 5 3-copy genes (including *Pcp4*, *Hmgn1*, *Cbr1*, and *Gabpa*), as well as BDNF, showed an interaction between genotype and treatment, suggesting a close relationship with this pathway.

Rescue of BDNF expression can also be obtained using green tea polyphenols (PGT) [42] and memantine [49] (see Tables 1 and 2). BNDF level rescue is associated with rescue of learning impairments, and thus plays a critical role in our understanding of DS and its potential therapies.

Regulation of the glutamate receptor, NMDAR, may be altered by several genes of HSA21, namely, through the calcineurin pathway. MK-801, a noncompetitive antagonist of NMDAR, may rescue memory retention, in particular, during aging. Locomotor activity of Ts65Dn and TS1Cje was evaluated in relation to different doses of MK-801 which block this receptor with a high affinity [52]. It was given at a dose leading to the same level of induced locomotion in the two strains. Proteins fractions (nuclear, cytosolic and membranous) of hippocampus and cortex were analyzed for their level in phosphorylation for proteins belonging to the Mapk pathway and for Tiam1, Itsn1, and Dyrk1a. Overexpression of these proteins was observed in Ts65Dn and Ts1Cje. Interestingly, a partial decrease in *Dyrk1a* and modified phosphorylation of MAPK proteins was observed in a genotype-specific pattern, suggesting that the genes responsible are at different locations on the trisomic segments [52, Table 2]. Interestingly MK-801 and memantine restore the phospho-mTOR level in Ts1Cje hippocampal dendrites [113]. But it is still to be proved that such treatment will benefit to the Ts65Dn memory impairment [111].

As an noninvasive approach, “environmental enrichment” that combines sensorimotor to social stimulations, may impact at the behavioral and molecular levels [156, 157]. Standardized methods (starting age, type of stimulation) may be needed to compare the changes observed and help understand why it benefits preferentially to Ts65Dn females.

Finally, molecular and cellular analyses in DS mouse models and DS brains show a clear correlation, though brain regions may vary in their specific features, confirming the utility of mouse models of DS for testing therapeutic treatments [158]. The number of therapeutic approaches in DS mouse models is rapidly increasing, with accompanying tests for behavioral rescue. However, little is known about the molecular and cellular consequences of these treatments; assessing these consequences will be crucial for future research and for any potential translation into the clinic.

## Figures and Tables

**Table 1:** Significant quantitative transcript variations observed in the DS mouse models (trisomics and transgenics, see Figure 1). Results are classified from top to bottom with increasing age of the mice studied: age in embryonic days (E), postnatal days (d) and month (m). Names in bold for genes present in 2 copies. Transcriptome methods used: C (cDNA arrays); N (Northern); M (microarrays); Q (quantitative-RT-PCR); R (RT-PCR). Gene names are indicated according to gene nomenclature (Gene Cards: http://www.genecards.org/).

Brain structures	Models	Age	Up	Down	Method	Additional comment	Target	Rescue	References
E11–E13 telencephalon, mesencephalon + diencephalon	TgYAC152F7	E11.5, E12.5, E13.5	Dyrk1a		Q, M	Dysregulation of the Rest pathway			[37]

Embryonic, brain hemispheres, cerebellum	Ts1Cje; TgPCP4	E11.5, E14.5, 4 m	Pcp4		Q				[38]

Embryonic total	Tc1	E14.5	—		R	Expression human genes			[32]

Embryonic	mBACTgDyrk1a	E14.5	Dyrk1A		Q				[25]

Total brain	Ts1Cje	birth	mean: 1.435		M				[39]

Cerebellum	Ts1Cje	birth	**Ptch**, **Shh**		M				[26]

Cortex, brain	Ts65Dn	8 d	**Vip**, **Vipr1**		Q				[40]

Total brain	Ts65Dn	1 m	62% of 3-copy genes		Q				[18]

Hippocampus, frontal cortex, substantia nigra	Ts65Dn	78–92 d	Kcnj6		Q				[41]

Hippocampus	TgYAC152F7	3 m	Dyrk1a	**Bdnf**,** Trkb**	Q		Dyrk1a	Bdnf, Trkb	[42]

Hippocampus	TS65Dn	3 m	Gart, Ifnar2, Kcnj6, Itsn1, Hcls, Sod1		M		Gabra5	Bdnf	[43]

Cerebellum	Ts65Dn	3-4 m	range (0.84–2.93); mean 1.45		M				[44]

Cortex, midbrain, cerebellum	Ts65Dn	4 m	mean: 1.63, 1.3, 1.37		C, M				[17]

Forebrain	Ts65Dn	4 m	App, Sod1, **ApoE**		N				[45]

Hippocampus (rescue), prefrontal cortex	Ts65Dn	5-6 m	mir155, mir802, **Mef2c**	**Creb1**, **Mecp2**	Q		mir-155, mir-802	Mecp2, Mef2c, Creb1	[46]

Brain hemispheres	Ts65Dn	4 to 12 m	App, Sod1, Dyrk1a		Q	increase with age			[21]

Brain	Ts65Dn	6-7 m		**Gfap**	Q				[47]

Hippocampus, cortex, raphe nuclei	Ts65Dn	9.5 m	**Vip**, **Vipr1**		Q				[48]

Hippocampus	Ts65Dn	10 m		**Bdnf**	Q		Nmdar	Bdnf	[49]

Total brain	Ts65Dn	11 m	47% of 3-copy genes		Q				[18]

Hippocampal CA1	Ts65Dn (m + f)	12–24 m	**Htr2c**	**Cdk5**, **Ntf3**	Q				[50]

Medial septum, hippocampus	Ts65Dn	18 m	App		Q				[51]

**Table 2:** Molecular changes observed in DS mouse models: Proteome.

Brain structures	Models	Age	Up	Down	Method	Additional comment	Treatment	Target	Rescue	References
Embryo E11; E14 SNC	Ts1Cje; TgPCP4	E11; E14	Pcp4, **Tubb3**, **Map2c**;** Calb2**		W, I					[38]

Neonatal brain	Ts65Dn	P0	**Map2**, **Ntf3**	**Syp**	W, I					[71]

Cortex	mBACTgDyrk1a	P0	Dyrk1a, **Gap43**		W					[25]

Hippocampus	Ts65Dn	P2	**Ptch1**		I					[72]

Hippocampus	Ts65Dn	P25	Kcnj6	**Gabbr2**	W					[73]

Medial septum	Ts65Dn	P2–20 m		**p75Ngfr** (6 m)	I					[45]

Thalamus, medulla oblongata	Ts65Dn	1 m	**Gabbr2**		W					[74]

Hippocampus	Ts65Dn	15–45 d		neuron, glia	I		fluoxetine		neurogenesis	[75]

Brain	Ts65Dn	49–66 d		**KIF17**	W	involv. NR2B transport				[76]

Hippocampus	Ts65Dn	2–4 m	**P** **(CaMKIIa, ** **AKT)**, **Glur1**, **p(Ser831)-** **Glur1**	**pERK**	W					[77]

Hippocampus, frontal cortex	Ts65Dn	80 d	Kcnj6, **Kcnj3**		W					[41]

Brain	Ts65Dn	3–5 m	App, Synj1		W					[54]

Brain	TgSYNJ1	3–5 m	Synj1		W					[54]

Cortex	mBACTgDyrk1a	3 m	Dyrk1A, **Ccnd1**, **Syp**, **Map2**		W					[25]

Hippocampus	Ts65Dn	1 m, 4 m, 12 m	App (12 m)		W		RS86 (agonist)	Chrm1	App increase (12 m) in Ts and 2N	[78]

Basal forebrain, hippocampus, paraventricular nucleus	Ts65Dn	3 m		**Nos1** in MSN, DB, PVN	I					[79]

Brain	Ts65Dn, TgSYNJ1	3–5 m	**PtdInsP**	**Ptdlns** **(4,** **5)P2**	E		Synj1 gene copy		yes	[54]

Hippocampus	Ts65Dn	4 m	**Ntf3**, **Cdk5**	**Syp**	W					[71]

Brain, hippocampus, cortex, striatum	Ts65Dn	4–12 m	App, Sod1, Dyrk1A, sAPP-alpha and -beta (12 m)		W					[21]

Brain hemispheres, cerebellum	Ts1Cje; TgPCP4	4 m	Pcp4		W, I					[38]

Somatosensory cortex	Ts65Dn	4-5 m	**Syp**, **Gad67**, **Calb**, **Calb2**, **Parv**		I					[80]

Brain hemispheres	Ts65Dn	5–12 m	increase with age: App, Sod1		W					[21]

Hippocampus (rescue), prefrontal cortex	Ts65Dn	5-6 m	**Mef2c**	**Creb1**, **Mecp2**	W					[46]

BFCN, hippocampus	Ts65Dn, Ts1Cje	6 m, 12 m	App, **Vchat** **hipp**. **termini**	**Ngf transport**	W, I	App copy numb. dep.				[81]

Hippocampus	Ts65Dn	7-8 m	Tiam1, Dyrk1a		W		MK801 ip	Nmdar	no	[52]

Cortex	Ts65Dn	7-8 m	Tiam1, Itsn1, Dyrk1A, **p(AKT**, **ERK**, **GSK3b)**		W		MK801 ip	Nmdar	Dyrk1a, **pERK1, 2**	[52]

Hippocampus	Ts1Cje	7-8 m	Dyrk1a; **pERK1,2**		W		MK801 ip	Nmdar	Dyrk1a; **pERK** **1,** **2**	[52]

Cortex	Ts1Cje	7-8 m	Itsn1, Dyrk1a, **p(AKT**, **ERK**, **GSK3b**)		W		MK801 ip	Nmdar	Dyrk1a	[52]

Medial septum, hippocampus	Ts65Dn ( m + f)	7–18 m	**microglia (CD45+)**	**Calb1 (hippo)**	I		minocycline (7–10 m)	inflammation	CD45, Calb1	[82]

Hippocampus, medial septum, locus coerelus	Ts65Dn	10 m		**Calb1 (H)**, **Chat (MS)**, **Th (LC)**	I		memantine (4–10 m)	Nmdar	no	[49]

Hippocampus, olfactory bulb, frontal cortex, cerebellum	Ts65Dn	10–19 m	**Chat (10** **m**, **all ages in cerebellum)**, **Glul (19** **m)**	**Chat in medial septum**, **AChe**	A					[83]

Medial septum BFCN	Ts65Dn	12 m		**p75Ngfr**	I					[84]

Hippocampus	Ts65Dn	12 m	**Chat**		A					[78]

Medial septum	Ts65Dn	12 m		**Chat**	I					[78]

Cerebellum	Ts65Dn	10–12 m	**Gfap**, **Sap/Jnk activation**		I, W	axonal damage				[85, 86]

Hippocampus, cingulate cortex	Ts65Dn/Ts1Cje	12–15 m	**Map2/ no**		I					[87]

Medial septum	Ts65Dn	18 m		**Chat**	I		Ngf infusion	Ngf transport	number and size	[84]

Fronto-parietal cortex, hippocampus	Ts65Dn	19 m	**Gfap**		W					[83]

Hippocampus, frontal cortex	TghAPP	24 m	Abeta 42, **UPS**	**alpha-**, **beta-secretase**, **Ngf**	E		RS86 (agonist)	Chrm1	Ngf, Abeta 42 increase (no)	[88]
